# Digital modeling of the gallbladder for revealing microanatomical features and optimizing surgical approaches in gallbladder-preserving cholelithotomy

**DOI:** 10.3389/fphys.2025.1710325

**Published:** 2026-01-14

**Authors:** Xiangtian Li, Xiyin Ye, Peng Yang, Haopeng Wu, Tingyu Fan, Zhaoming Xiao, Qiyang Jiang, Zheyang Lin, Shasha Peng, Tingyi Huang, Xiaohui Feng, Yuan Liang, Yu Wang, Jun Ouyang, Jingxing Dai, Sangui Wang

**Affiliations:** 1 The Second Clinical Medical College, Southern Medical University, Guangzhou, China; 2 Dongguan Institute of Hepatobiliary Diseases, Dongguan Nancheng Hospital, Dongguan, China; 3 The First School of Clinical Medicine, Southern Medical University, Guangzhou, China; 4 Guangdong Provincial Key Laboratory of Digital Medicine and Biomechanics and Guangdong Engineering Research Center for Translation of Medical 3D Printing Application and National Virtual and Reality Experimental Education Center for Medical Morphology (Southern Medical University) and National Key Discipline of Human Anatomy, School of Basic Medical Sciences, Southern Medical University, Guangzhou, China; 5 School of Health Management, Southern Medical University, Guangzhou, China; 6 School of Basic Medical Sciences, Southern Medical University, Guangzhou, China; 7 Department of Ultrasound Medicine, Xiangyang Central Hospital, Hubei University of Arts and Science, Xiangyang, China

**Keywords:** gallstones, minimally invasive surgery, precision slicing, surgical approach, three-dimensional reconstruction, vascular distribution

## Abstract

With the advancement of minimally invasive techniques and the reevaluation of surgical indications, gallbladder-preserving cholecystolithotomy is anticipated to serve as an alternative to cholecystectomy in specific cases. The choice of surgical incision is critical for optimizing gallbladder function preservation, which is a pivotal factor influencing the prognosis of gallbladder-preserving cholecystolithotomy. Consequently, a comprehensive understanding of the distribution of microstructures, including gallbladder blood vessels and nerves, is of substantial significance. For this study, we selected the gallbladders of healthy four-year-old children as our subjects. Gallbladder specimens were dehydrated, paraffin-embedded, and serially sectioned into hundreds of 4-μm-thick slices. Sections were selectively stained with distinct protocols—hematoxylin and eosin (H&E) and anti-tyrosine hydroxylase immunohistochemical staining—followed by sequential numbering. Digitized sections were reconstructed into three-dimensional models (3D) using computational software. The resultant 3D gallbladder models achieved a resolution threshold of <20 μm, enabling visualization of microvascular and neural structures. Independent and integrated analyses of the modeled cystic arteries, veins, and sympathetic neural networks revealed two superficial arterial trunks and one deep branch originating from the superficial division of the cystic artery, with their interactive patterns defining nutrient-supplying territories. Further mapping of microvascular and neural trajectories within the digital models identified a minimally function-disruptive surgical incision site, diverging from conventional fundal incision approaches for gallstone extraction. This approach offers a 3D visualization framework to enhance pathological slice interpretation—thereby facilitating histopathological diagnosis—and is proposed as a novel surgical route for gallbladder-preserving cholelithotomy.

## 1 Introduction

Gallstones are among the most prevalent gastrointestinal diseases worldwide (Portincasa et al., 2006), and cholecystectomy is regarded as the standard surgical treatment for this condition (Qu et al., 2020). A critical step in the procedure is the identification and ligation of the cystic artery within Calot’s triangle (Abdalla et al., 2013). However, as our understanding of gallbladder function deepens and the incidence of gallbladder diseases in younger populations rises, cholecystectomy may not universally apply as a solution (Sicklick et al., 2005). In specific cases, gallbladder-preserving cholelithotomy may provide a more favorable prognosis (Tang et al., 2024).

Consequently, we are dedicated to investigating the optimal approach for gallbladder-preserving cholelithotomy, focusing on avoiding significant blood vessels and nerves to minimize damage to the gallbladder and enhance the preservation of its function. Specifically, preservation of the intact vascular supply prevents intraoperative hemorrhage and ischemic necrosis (Pes et al., 2023), while sparing the autonomic neural network maintains gallbladder contractility, thereby reducing the risk of biliary stasis and subsequent stone recurrence (Housset et al., 2016; Venneman and van Erpecum, 2010). To achieve this goal, it is essential to construct a three-dimensional physiological model of the gallbladder and to develop a comprehensive understanding of the cystic artery’s course, the internal structure of the gallbladder, and the distribution and vascular density of its branches (Krishnamurthy et al., 1981; Schiewe et al., 2025).

Current technologies for constructing 3D gallbladder models primarily encompass radiological imaging (ultrasound, computed tomography (CT), magnetic resonance imaging (MRI)) (Morgan et al., 2018; Martins et al., 2025), tissue optical clearing, and digitized section-based 3D reconstruction—each with distinct functional applications (Martins et al., 2025; Ye et al., 2023). Advantages of radiological models include rapid spatial distribution mapping, which is particularly effective for detecting anatomical variations; low cost; and intraoperative augmented reality (AR) navigation capability for optimal surgical pathway selection (Zhao et al., 2022). However, limitations include insufficient resolution for visualizing microvascular trajectories. Although computed tomographic angiography (CTA) partially displays vasculature (Yang et al., 2014), contrast agent constraints restrict visualization to main trunks and partial branches (Rojo Ríos et al., 2023). Tissue optical clearing also faces inherent limitations such as antibody permeability dependency, prolonged processing (1–2 months/sample) (Kiemen et al., 2023), and unstable imaging outcomes. Cost escalates significantly with increased sample volume, hindering human organ studies.

**FIGURE 1:**
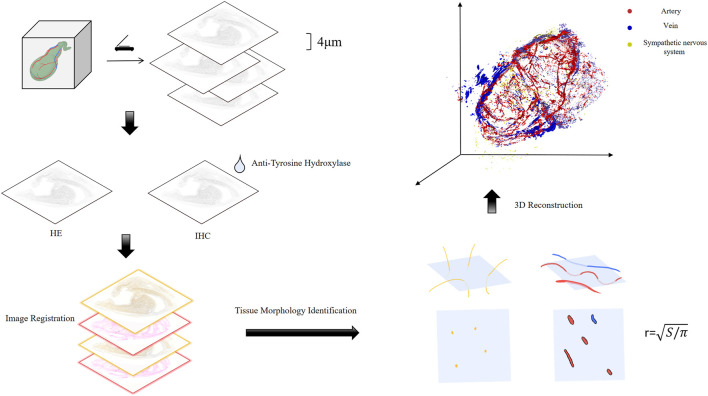
Workflow for digital gallbladder model construction.

In contrast, three-dimensional reconstruction technology based on digital slices is not constrained by tissue characteristics. This versatility has been demonstrated in previous studies visualizing the spatial heterogeneity of pancreatic ductal adenocarcinoma (Kiemen et al., 2022), the intricate bile canaliculi and sinusoidal networks in the liver (Segovia-Miranda et al., 2019), and the microvascular architecture of the brain (Gijtenbeek et al., 2005). Beyond enabling precise spatial localization of microstructures, this technology also permits flexible adjustment of slice thickness according to research requirements, thereby optimizing the resolution of gallbladder microstructures. In this study, we aimed to reconcile high-precision vascular visualization with cost-efficiency by adopting digitized sectioning integrated with high-resolution scanning, which resulted in a gallbladder model with a resolution of 4 μm (Figure 1). Utilizing this model, we quantitatively analyzed the neurovascular distribution to propose optimal surgical incisions that minimize damage to the gallbladder’s critical blood vessels and nerves.

## 2 Materials and methods

### 2.1 Gallbladder tissue section preparation

Gallbladder specimens were procured from a 4-year-old female victim of accidental death with no documented pathology. Tissues were fixed in 4% paraformaldehyde (PFA) for 48 h, dehydrated through a graded ethanol series, treated with 1:1 anhydrous ethanol-xylene mixture for 15 min, cleared in xylene I (15 min) and xylene II (15 min) to achieve transparency, and infiltrated with xylene-paraffin mixture (1:1) for 15 min. Complete infiltration was ensured by immersion in paraffin I (65 °C, 1 h), paraffin II (65 °C, 21 h), and paraffin III (65 °C, 1 h). After cooling, serial sections were cut at 4-μm thickness.

To achieve simultaneous visualization of distinct anatomical systems within the same spatial coordinate system while maintaining cost-efficiency, a strategic grouping approach was employed. A total of 224 serial sections were divided into two interleaved sets—Set 1: odd-numbered sections (1, 3, 5,...) and Set 2: even-numbered sections (2, 4, 6,...). This alternating arrangement leveraged the spatial continuity of adjacent slices to minimize alignment errors during 3D reconstruction. Set 1 was subjected to conventional H&E staining to delineate vascular morphology, while Set 2 underwent immunohistochemistry (IHC) using anti-tyrosine hydroxylase (TH) antibody (ab137869, Abcam, 1:200) to specifically target sympathetic nerves. All stained sections were mounted with neutral gum.

### 2.2 Digitalization of gallbladder sections

**FIGURE 2:**
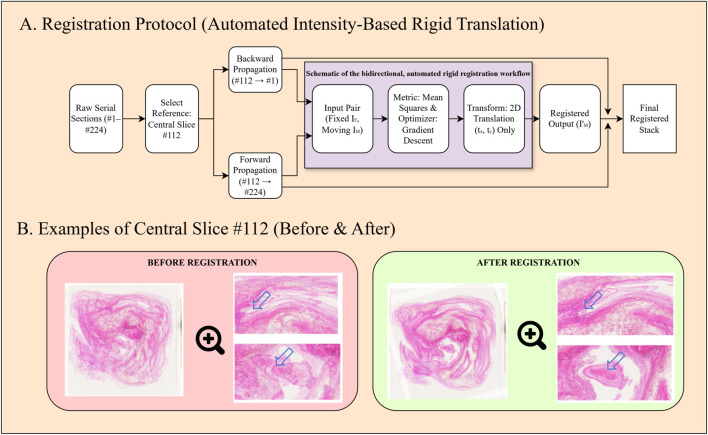
**(A)** Schematic illustration of the image registration workflow. **(B)** Comparison of pre- and post-registration alignment. The post-registration images demonstrate significantly improved structural continuity and spatial consistency.

To achieve spatial correspondence across the serial sections, automated image registration was performed using the SimpleITK library in Python (Figure 2). An intensity-based rigid registration algorithm was employed, utilizing Mean Squares as the similarity metric and a Regular Step Gradient Descent optimizer. The transformation was constrained to 2D translation (rigid transformation) to correct positional offsets (t_x_, t_y_) while strictly preserving the original rotation and scale of the tissue structures. The central section (#112) served as the initial reference, and registration propagated bidirectionally through the stack. Registration quality was automatically monitored using the correlation coefficient, ensuring robust alignment without the observer variability associated with manual landmark placement.

### 2.3 Construction of digital gallbladder models

Following the previously described protocols, pediatric gallbladder specimens were subjected to sequential fixation, embedding, sectioning, H&E/TH-IHC staining, and digitization. Subsequently, 3D segmentation and reconstruction were performed using Mimics 21 (Materialise, Belgium).

Digitized images were imported into Mimics and spatially calibrated (Software readout 60.05 mm = Physical truth 1600 μm; pixel size: 0.0266 mm) to ensure dimensional accuracy. Given the complex morphology and varying contrast of the microvasculature, a manual segmentation protocol was employed to ensure topological precision. To maintain consistency, the segmentation environment was standardized: image contrast was enhanced using a fixed window/level setting, and segmentation was executed slice-by-slice using a pixel-perfect brush tool (1–3 pixels diameter) under high magnification.

Upon completion of segmentation, 3D models were generated. To minimize global construction variability and observer bias inherent in manual protocols, a “segmented calculation and summation” strategy was applied. Furthermore, the reproducibility of this manual construction was rigorously validated. As detailed in Table 1, repeated morphometric measurements (n = 3) of the inner and outer diameters at standardized landmarks demonstrated minimal variability (Standard Deviation <10% of the mean diameter), confirming the robustness of the manual delineation process.

**TABLE 1:** Morphometric dimensions of the main neurovascular structures (Mean ± SD).

Vessel	Diameter type (μm)	Point 1 (proximal)	Point 2	Point 3	Point 4	Point 5 (distal)
Artery α	Inner	232.3 ± 8.2	152.7 ± 14.2	159.2 ± 7.4	74.4 ± 6.2	75.0 ± 2.2
	Outer	325.2 ± 14.3	264.0 ± 8.8	252.0 ± 2.9	176.2 ± 2.6	143.3 ± 5.1
Artery β	Inner	173.0 ± 7.8	152.7 ± 7.8	88.7 ± 11.3	108.0 ± 10.8	95.9 ± 3.7
	Outer	280.1 ± 8.5	234.8 ± 5.6	180.7 ± 2.3	214.6 ± 11.5	163.9 ± 9.8
Artery γ	Inner	64.7 ± 8.7	72.2 ± 4.9	56.3 ± 4.2	47.7 ± 2.8	46.2 ± 7.0
	Outer	172.3 ± 13.1	163.1 ± 11.6	145.6 ± 2.8	110.0 ± 37.1	150.5 ± 2.0
Vein δ	Inner	192.9 ± 14.1	137.1 ± 11.6	86.8 ± 2.2	197.4 ± 9.8	97.8 ± 4.2
	Outer	333.1 ± 14.4	283.9 ± 20.4	232.6 ± 6.6	306.7 ± 15.8	202.9 ± 10.0

### 2.4 Sample processing for transmission electron microscopy

Gallbladder samples underwent primary fixation in 2.5% glutaraldehyde/0.1M phosphate buffer (pH 7.0, ≥4 h) followed by three 15-min buffer rinses. For secondary fixation, 1% OsO_4_ in identical buffer (1–2 h) was used, with subsequent rinses. Dehydration progressed through graded ethanol (30%, 50%, 70%, 80%; 15 min/step) and acetone (90%, 95%; 15 min/step) series, culminating in two 20-min absolute acetone incubations. Resin infiltration was initiated in 1:1 acetone/Spurr resin (1 h, RT), transitioned to a 1:3 mixture (3 h), followed by pure resin (overnight). Samples were polymerized in molds (70 °C, ≥9 h), sectioned at 70–90 nm (Leica EM UC7), dual-stained with uranyl acetate (10 min) and lead citrate (5–10 min), and imaged on a Hitachi H-7800 transmission electron microscope (TEM) (80–100 kV).

### 2.5 Specimen casting

Arterial perfusion was initiated with 25 mL of low-viscosity red acrylic resin (Zidan et al., 2020) injected through the celiac trunk. Upon resin outflow from the superior mesenteric artery, the inferior mesenteric arterial cannula was clamped. Perfusion continued until filling of transverse colic/gastroepiploic/cystic arteries and hepatic speckling, followed by 30-min stabilization. High-viscosity resin was supplemented into celiac and superior mesenteric arteries (20 mL each). Specimens were polymerized at 40 °C for 24 h. For biliary perfusion, 25 mL green resin was utilized via the cystic duct until gallbladder/hepatic duct distension. Specimens in anatomical position underwent corrosion in 37% HCl (3–4 days), followed by high-pressure water rinsing, 1% ammonia neutralization (24 h), 5%–10% H_2_O_2_ bleaching (24 h), and thorough washing with water.

### 2.6 Comparative analysis of vasculature density in left-right gallbladder regions

A reference line bisecting each segmented section along the longitudinal axis was delineated. Images were batch-imported into Adobe Photoshop CC 2019 (Adobe Inc., USA). Using the Slice Tool, sections were divided along reference lines into left/right halves via: *Slice Tool → Right-click → Divide Slice → Vertically, Number of Slices: 2.* Slices were then exported as individual TIFF files. Files were batch-processed in ImageJ v1.53 (NIH), and vasculature density was quantified. Given that the left and right regions were derived from the same tissue section, statistical significance was assessed using a Paired t-test (α = 0.05) to account for the matched nature of the samples. Data visualization was performed using GraphPad Prism v10.1.2 (GraphPad Software, USA).

### 2.7 Quantification of gallbladder vascular diameters

Quantification of gallbladder vascular diameters was performed in accordance with (Łabętowicz et al., 2025; Thiyagarajah et al., 2025). Vascular locations were identified on 2D sections by cross-referencing the reconstructed 3D gallbladder model. Five measurement points were systematically selected per vessel. Lumen and external diameters were measured at designated points using Mimics’ integrated measurement tools, with triplicate measurements per point averaged. For cross-sections, lumen area was measured for radius calculation via Equation 1. Longitudinal sections permitted direct measurement of the diameter. Scaled dimensions were derived using embedded scale bars. Data visualization was performed in GraphPad Prism v10.1.2 (GraphPad Software, USA).

$$r=\sqrt{\frac{S}{\pi }}$$ (1)

## 3 Results

### 3.1 Trajectories of principal gallbladder vasculature

**FIGURE 3:**
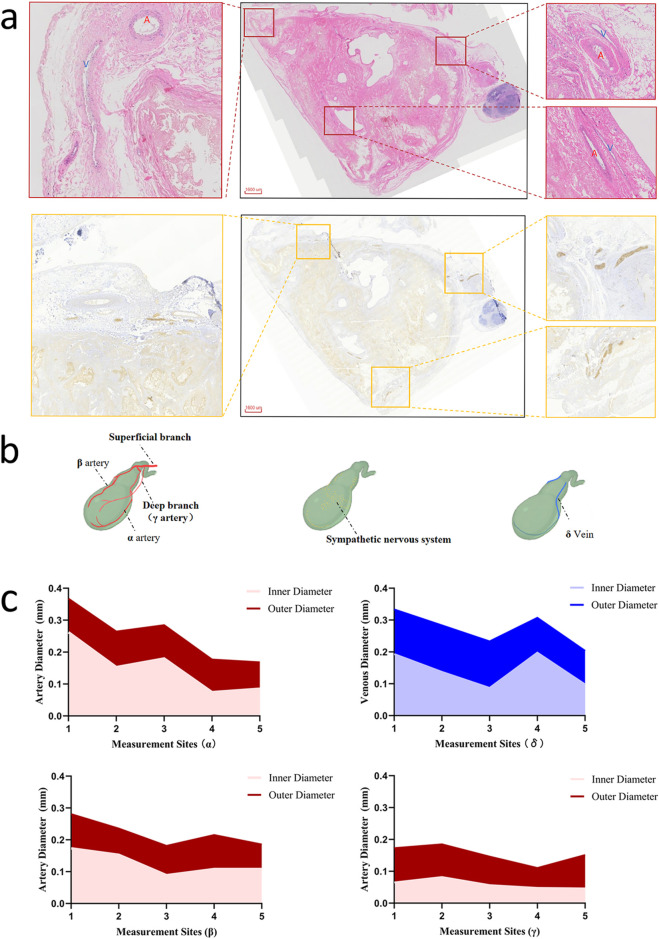
**(a)** H&E staining and immunohistochemistry (IHC) identification of arteries, veins, and nerves in gallbladder sections. **(b)** Spatial distribution of vasculature and neural networks in the digital gallbladder model. **(c)** Quantification of the diameter of pericholecystic main vascular trunks.

Through H&E staining and tyrosine hydroxylase immunohistochemistry (TH-IHC) for sympathetic nerve specificity, arteries, veins, and sympathetic nerves were annotated using Mimics software. H&E staining distinguished arteries by their circular luminal profiles, contrasting with the collapsed or irregularly elliptical venous structures consistently observed in adjacent vascular pairs (Figure 3a). Three-dimensional reconstructions delineated the vascular and neural distributions (Figure 3b), revealing superficial cystic artery branches bifurcating into α- and β-arteries traversing the gallbladder sides. The α-artery demonstrated extensive branching and anastomosed with the deep γ-branch, establishing nutrient-supplying collaterals. A single dominant δ-vein paralleled the α-artery toward the fundus. Sympathetic nerves exhibited a diffuse distribution without fasciculated bundles and were predominantly localized in the neck and body regions.

Quantitative morphometric analysis at five standardized points along the longitudinal axis revealed distinct dimensional hierarchies (Table 1). The α-artery served as the dominant arterial trunk, tapering from a proximal outer diameter of 325.2 ± 14.3 μm to a distal 143.3 ± 5.1 μm. The β-artery was comparatively smaller, with a proximal outer diameter of 280.1 ± 8.5 μm. The deep γ-branch represented the finest vascular structure identified, exhibiting a minimum outer diameter of 110.0 ± 37.1 μm (Point 4) and an inner diameter as narrow as 46.2 ± 7.0 μm at the distal tip (Point 5). The δ-vein showed a caliber comparable to the α-artery, with a proximal outer diameter of 333.1 ± 14.4 μm (Figure 3c).

### 3.2 Visualization of microscopic gallbladder structures

**FIGURE 4:**
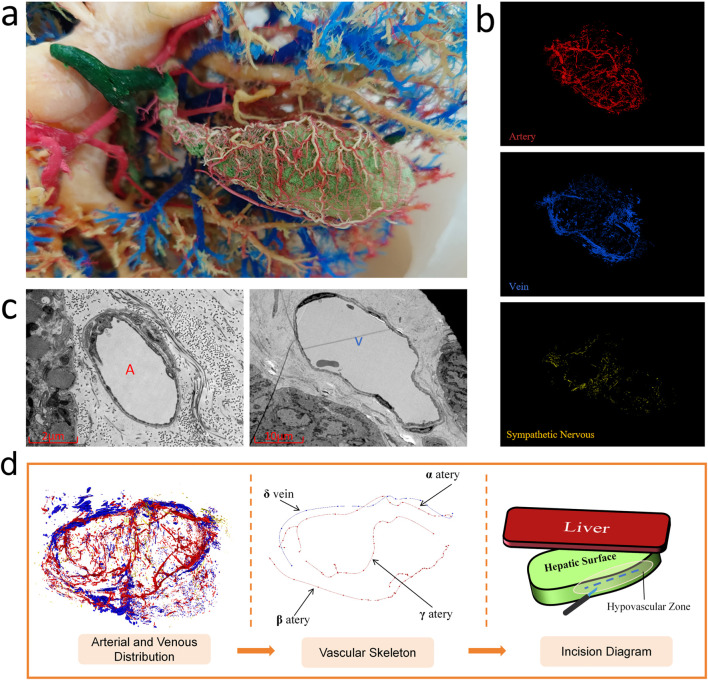
**(a)** Macroscopic view of the arteriovenous-perfused gallbladder specimen. **(b)** Three-dimensional reconstruction of micro-arteries, micro-veins, and sympathetic nerves. **(c)** Scanning electron microscopy (SEM) of intramural microvasculature. **(d)** Vascular skeleton map highlighting the major vascular trunks and the proposed optimal surgical incision within the medial avascular safety zone.

Dye perfusion of gallbladder specimens revealed numerous micro vessels distributed peripherally, with densely aligned arteriovenous pairs and extensive intervascular anastomoses (Figure 4a). Conventional gallbladder-preserving cholelithotomy via fundal incision risks inadvertent transection of microvasculature and functional nerves (Tan et al., 2013). Limitations of traditional dye perfusion include restricted penetration due to high viscosity, which impairs visualization of sub-20-μm vessels, and lack of quantitative digital analysis. Three-dimensional reconstructions using Mimics software delineated microvascular and sympathetic neural networks (Figure 4b), with fusion of topologically correlated models enhancing spatial relationship comprehension. Scanning electron microscopy (SEM) at ×2,000 magnification further enabled visualization of murine gallbladder microvasculature (Figure 4c), achieving superior vascular discrimination comparable to H&E staining at the histological level. To precisely delineate the spatial topology of the neurovascular network, the reconstructed 3D digital model was processed to generate a vascular skeleton map (Figure 4d). This skeletonization filtered out volumetric noise, clearly highlighting the main arterial trajectories, particularly the course of the β-artery along the medial aspect. Based on this topology, a specific ‘safety window’ was identified on the medial aspect of the gallbladder body (facing the stomach). To strictly preserve the blood supply, the optimal surgical incision was strategically designated just distal (superior) to the trajectory of the β-artery. By placing the incision in this specific medial avascular zone, the procedure effectively avoids the β-artery while ensuring safe access near the hepatic margin.

### 3.3 Optimal surgical approach assessment for calculous gallbladders

**FIGURE 5:**
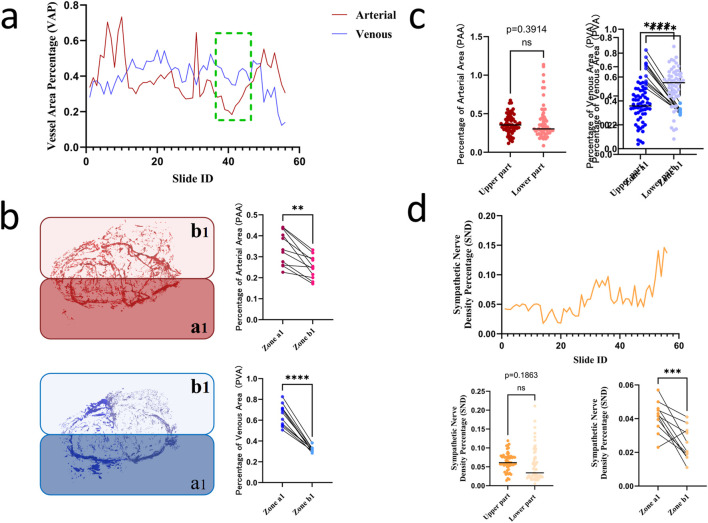
**(a)** Optimal incision level across gallbladder sections; **(b)** Comparative vascular density in a1 *versus* b1 territories (*P* < 0.005); **(c)** Comparison of vascular density between superior and inferior segments (arteries *P* = 0.3914, veins *P* < 0.0001); **(d)** Quantification of sympathetic nerve density.

Following arterial/venous annotation in Mimics, the venous density per section was quantified (Table 2). The results revealed that sections 36–46 exhibited minimal vascular density (Figure 5a), with the arterial area fraction nadir at 0.1835%, indicating reduced hemorrhagic risk for incisions in this zone. This section range was subdivided into the following hemigallbladder regions: α-artery territory as a1 and β-artery territory as b1. Regional density analysis demonstrated significantly higher vascularity in a1 than in b1 (*P* < 0.005; Figure 5b). Vertical partitioning showed comparable arterial density (*P* = 0.3914), but significantly lower venous density in superior *versus* inferior segments (*P* < 0.0001; Figure 5c), indicating that superior incisions minimize vascular compromise. Quantification of tyrosine hydroxylase-positive nerves (Figure 5d) revealed denser sympathetic innervation in superior sections, predominantly associated with the deep γ-branch, while the b1 territory exhibited relatively lower neural density.

**TABLE 2:** Percentage distribution of arterial and venous densities in gallbladder H&E-stained sections.

Slid ID	Arterial (%)	Venous (%)
1	0.3365	0.2805
2	0.3915	0.37
3	0.3465	0.374
···		
36	0.304	0.4735
37	0.281	0.455
38	0.2885	0.4445
39	0.2115	0.392
40	0.205	0.385
41	0.1835	0.3525
42	0.232	0.349
43	0.2545	0.426
44	0.2885	0.443
45	0.3365	0.427
46	0.355	0.374
···		
55	0.3525	0.1865
56	0.305	0.123
57	0.3795	0.14

## 4 Discussion

While Laparoscopic Cholecystectomy (LC) remains the gold standard for gallstone disease, it is not without long-term sequelae. A prospective multicenter study (Wennmacker et al., 2017) indicates that up to 41% of patients may experience persistent abdominal symptoms following organ removal. This concern is particularly amplified in pediatric populations—such as the 4-year-old donor in our study—where organ preservation is prioritized to support long-term development. Regarding the comparison with radical resection, recent comprehensive clinical analyses regarding pediatric cholelithiasis and surgical outcomes (Akhtar-Danesh et al., 2018; Sun et al., 2025) indicate that for selected patients, preserving strategies should be carefully weighed against cholecystectomy. In this context, our anatomical study aims to provide a precise safety map to further optimize this organ-preserving approach by minimizing neurovascular injury.

Conventional clinical focus centers on the cystic artery and its variants (e.g., duplicated arteries) (Zhu et al., 2023) in cholecystectomy contexts. In contrast, this study prioritizes mapping pericholecystic and intramural vascular patterns to optimize surgical approaches for gallbladder-preserving cholelithotomy (GPC). Digital modeling revealed that the pericholecystic arterial architecture comprises a deep branch (γ) and bifurcated superficial branches, namely, the right α-artery and left β-artery. Notably, the γ-branch anastomoses with the α-artery, while the δ-vein courses parallel to the α-artery. Sympathetic nerves exhibit diffuse intramural distribution, with denser clustering in the neck and body regions.

Statistical analyses of vascular and neural densities across gallbladder sections identified the superior aspect and left-sided b1-territory as low-vascularity zones, with concomitantly reduced sympathetic innervation in the b1-territory. The branched architecture of the α-artery and its anastomosis with the deep γ-branch denote structural dominance over the β-artery, validating the b1-superior quadrant as the optimal incision site to minimize iatrogenic injury in gallbladder-preserving procedures.

Synthesizing these findings, we strongly recommend a left-biased longitudinal incision along the gallbladder-hepatic interface. This approach leverages proximity to the minimally branched β-artery to reduce major vasculature injury risk, while the inherent low microvascular density minimizes collateral damage. Furthermore, concomitant neural preservation maintains physiological contractile function (Zhang et al., 2014). Critically, maximal distance from the deep γ-branch further mitigates iatrogenic trauma, ensuring unimpaired nutrient delivery to the gallbladder parenchyma post procedure, thereby enhancing postprocedural recovery of contractile and absorptive functions (Zhang et al., 2017).

In contrast to our strategy, our topological analysis indicates that conventional fundal incisions traverse the anastomosis zone between the deep γ-branch and α-artery, thereby posing a significant risk of disrupting this vascular network. This vascular compromise could theoretically impair local perfusion, potentially affecting postoperative recovery. Consequently, our perspective advocates for an incision placement that respects this vascular anatomy, diverging from conventional gallbladder-preserving cholelithotomy utilizing fundal incisions (Costamagna et al., 2002). Concurrently, we pioneer a novel application methodology regarding the serial section-based modeling framework applicable to pathological tissues. This approach significantly enhances the delineation of tissue invasion patterns and spatial distribution profiles (Zhang et al., 2020), thereby refining pathological diagnostics with superior precision (Tan et al., 2016).

Future investigations should use electron microscopy to capture microscopic gallbladder vasculature, enabling construction of higher-fidelity digital gallbladder models. Advancing artificial intelligence (AI) technologies, particularly deep learning applications (Zhang et al., 2023), will significantly enhance identification efficiency for vascular and neural structures.

## 5 Limitations

Several limitations of this study must be acknowledged to avoid clinical overgeneralization. First, the 3D reconstruction was based on a single specimen from a healthy 4-year-old donor. While this provided a pristine, non-fibrotic baseline for mapping native neurovascular topology, it does not account for age-dependent vascular remodeling or inter-individual anatomical variations prevalent in the adult population. Second, the absence of pathological changes—such as chronic inflammation, fibrosis, and gallstone burden—limits the direct translatability of our findings to clinical cases where tissue architecture may be significantly distorted. Consequently, the neurovascular ‘safety zones’ identified herein should be interpreted as anatomical recommendations based on ideal topology rather than absolute surgical rules. Future studies incorporating adult cadaveric specimens and pathological samples are essential to validate the robustness of this incision strategy across diverse patient cohorts.

## Data Availability

The raw data supporting the conclusions of this article will be made available by the authors, without undue reservation.
